# Uterine angiomyofibroblastoma in a domestic cat: A need of precise diagnosis for proper perioperative management

**DOI:** 10.17221/88/2024-VETMED

**Published:** 2025-05-26

**Authors:** Tae-Un Kim, Jai Soon Park, Jae-Hyuk Yim, Woo Jun Kim, Sung Bin Hong, Yi-Rang Jung, Seong-Kyoon Choi, Su-Min Baek, Jin-Kyu Park

**Affiliations:** ^1^Department of Veterinary Pathology, College of Veterinary Medicine, Kyungpook National University, Daegu, Republic of Korea; ^2^Gumi Top Animal Medical Center, Gumi, Republic of Korea; ^3^Daegu Health College, Department of Companion Animal Health Management, Daegu, Republic of Korea; ^4^Core Protein Resources Center, Daegu-Gyeongbuk Institute of Science and Technology (DGIST), Daegu, Republic of Korea; ^5^Institute for Veterinary Biomedical Science, Kyungpook National University, Daegu, Republic of Korea

**Keywords:** α-SMA, desmin, feline, hemangiopericytoma, uterus

## Abstract

Angiomyofibroblastoma (AMFB) is rarely reported in humans as well as domestic animals, especially in the female genital tract. This painless tumour is characterised by the proliferation of spindle or ovoid tumour cells with myofibroblastic differentiation, which often cluster around thin-walled blood vessels. This report presents a case of feline AMFB for the first time. Preoperative abdominal radiography demonstrated an enlarged uterus with the dorsolateral displacement of the ovaries, and transabdominal ultrasonography showed an enlarged uterus with diffuse hypoechoic areas. The resected uterus showed a firm texture with a grey to brownish colour. A microscopic examination revealed proliferation of well-differentiated neoplastic spindle cells on a background of abundant fibrous stroma containing numerous blood vessels. Myxoid degeneration and vascular extravasation were not observed. The neoplastic cells were diffusely immunopositive for α-SMA and vimentin and focally positive for desmin (in the perivascular areas). AMFB is rare in veterinary medicine and the feline uterine AMFB observed in the present case had not been previously reported. Although previously reported as a safe, benign tumour, a surgical procedure of a large AMFB in small animals without proper diagnosis may become life-threatening.

Angiomyofibroblastoma (AMFB) is an unusual type of mesenchymal tumour with benign histological features (Laiyemo et al. 2005; Eckhardt et al. 2018). Commonly diagnosed in the lower genital tract of females, the tumour is located predominantly in the perineum, uterine cervix, or vulvovaginal area (Laiyemo et al. 2005; Qiu et al. 2014). Human AMFB is typically measured as less than 5 cm in size, while a maximum of 23 cm has been reported thus far (Eckhardt et al. 2018). AMFB is an unusual lesion in both humans and animals, where only 71 cases of human vulvar AMFB were reported until 2012 in English publications (Sims et al. 2012). According to a retrospective study involving 4 157 women who underwent urogynecological surgery, there were only three cases of AMFB (Liaci et al. 2017). AMFB has not been reported in veterinary medicine except for a single canine case reported in a retrospective study of perivascular wall tumours (Avallone et al. 2007). Local excision is the curative treatment for AMFB, which has an exceptionally low recurrence rate (Qiu et al. 2014; Shilpa et al. 2022). AMFB has been reported to be observed as a small, well-demarcated mass in the subcutaneous layer (Nagai et al. 2010; Wang et al. 2018). However, AMFB can occasionally reach large sizes, up to 20 cm, especially in the vulvovaginal area, pelvis, peritoneal cavity, or iliac fossa (Nagai et al. 2010; Qiu et al. 2014; Wang et al. 2018).

Uterine tumours are uncommon in cats, accounting for 0.29% of all feline neoplasms reported during a 9.6-year period in one report (Miller et al. 2003). Uterine tumours include adenoma, leiomyoma, fibroma, fibroleiomyoma, and their malignant counterparts (Miller et al. 2003; Agnew and MacLachlan 2017). Myometrial leiomyoma is the most common mesenchymal tumour in the uterus, characterised by interlacing muscle fibres (Agnew and MacLachlan 2017). Other mesenchymal tumours in the tubular tract include lymphosarcoma and lipoma within the broad ligament and ovarian bursa (Agnew and MacLachlan 2017). A case of AMFB in the feline uterus has not been reported in veterinary medicine, thus the procedures of the surgical and post-operative management have not been established.

However, AMFB, as in the present case, may be life-threatening without proper perioperative procedures due to its characteristics. The present report describes a case of feline uterine AMFB completely replacing the uterine structure, along with its clinical and histopathological characteristics.

## Case presentation

**Figure 1:**
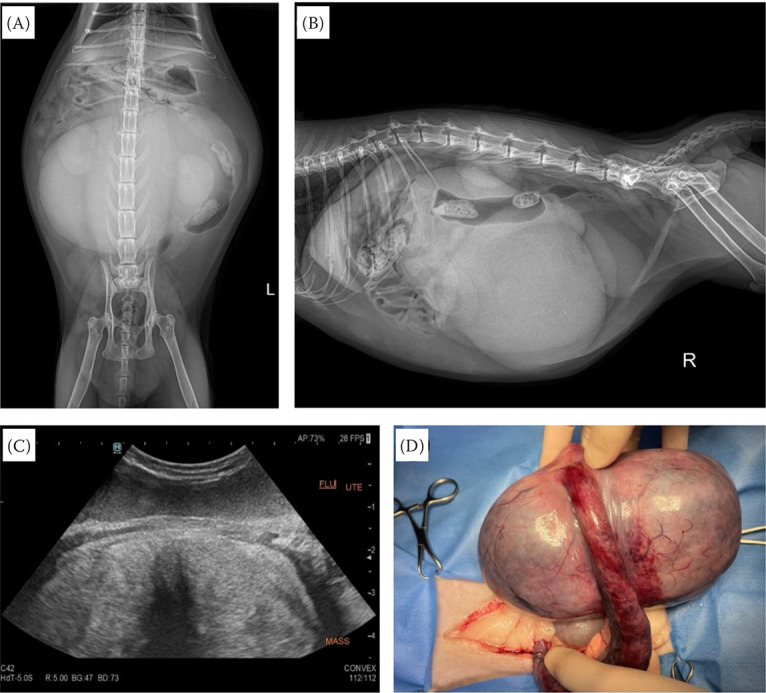
Clinical findings (A,B) Radiograph images of the abdomen. An enlarged uterus and displacement of the other organs were detected. (C) Ultrasonographic image of the enlarged uterus. (D) Surgical excision of the uterus

An 18-year-old unneutered female Korean domestic shorthair cat was admitted to a local hospital with abdominal distension. The patient showed anorexia along with severe hypothermia (35.5 °C, normal range: 38.3-39.2 °C). The complete blood cell counts and serum chemistry analyses showed the elevation of feline proBNP (552 pmol/l, normal range: 0–100 pmol/l), serum lactate (10.9 mmol/l, normal range: 0.5–2.5 mmol/l), and white blood cell counts including lymphocytes and neutrophils (47.91 × 10^9^/l, normal range: 5.5–19.5 × 10^9^/l). Abdominal radiography revealed the total enlargement of the uterus with the craniodorsal displacement of the small bowel and dorsolateral displacement of the ovaries (Figure 1A, B). Transabdominal ultrasonography also showed an enlarged uterus in the lower abdomen (Figure 1C). Uterine leiomyosarcoma or pyometra was suspected, and an immediate hysterectomy was performed (Figure 1D). The patient died of sudden cardiac arrest after ligation of the ovarian branch of the uterine artery.

**Figure 2:**
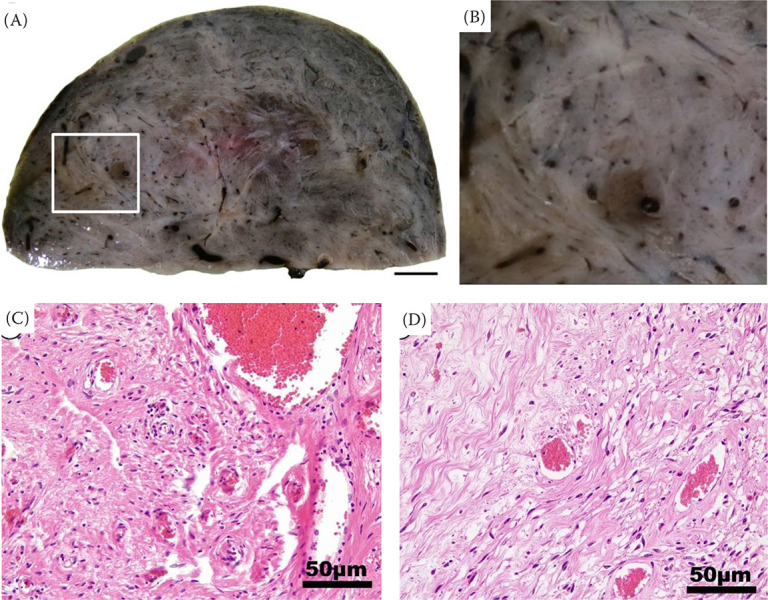
Gross and histopathological findings of angiomyofibroblastoma (A,B) Gross findings of the resected uterus. (A) Cut surface exhibiting a grey to brownish colour with haemorrhagic spots. Scale bar = 1 cm. (B) Numerous blood vessels were detected. (C,D) Microscopic findings, haematoxylin and eosin staining. (C) Numerous blood vessels were surrounded by neoplastic spindle cells, and perivascular cell clustering was observed. Scale bar = 50 μm. (D) The neoplastic spindle cells exhibited minimal pleomorphism and no mitotic figures and showed alternative hypercellular and hypocellular areas. Scale bar = 50 μm

The resected uterus was non-pedunculated and had a thick, firm texture with a size of 10 cm × 6 cm. The sample was immediately fixed in 10% neutral buffered formalin for a histopathological examination. The cut surface of the mass had diffused haemorrhagic foci with a grey to brownish colour (Figure 2A,B). There were no signs of cystic change or purulent exudates. After thorough processing, the sample was embedded in paraffin and sectioned at a 3 μm thickness for the microscopic and immunohistochemical analyses. The histopathological analysis revealed a spindle cell mesenchymal tumour, with alternating hypocellular and more cellular areas, on a background of abundant fibromyxoid stroma (Figure 2C). Numerous blood vessels with perivascular hyalinisation were surrounded by abundant fibromyxoid stroma lacking inflammatory cells (Figure 2D). The overall histology comprising the absence of pleomorphism and mitotic figures suggested that the mass was a well-differentiated benign neoplasm.

**Figure 3:**
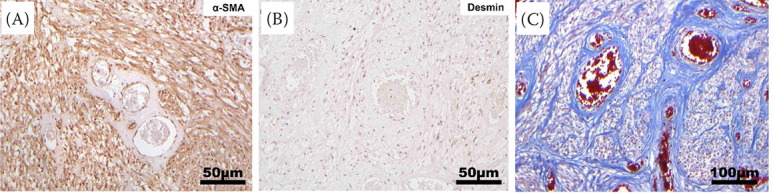
Immunohistochemistry and Masson's trichrome staining (A) Immunohistochemical labelling for alpha-smooth muscle actin. The neoplastic spindle cells were immunopositive to α-SMA. (B) Immunohistochemical labelling for desmin. In this area, the neoplastic spindle cells showed negative immunoreactivity to desmin. (C) Masson’s trichrome staining. The thick fibrous stroma surrounding the capillaries was stained blue. Scale bars = 50 μm

Immunohistochemistry was performed for the differential diagnosis of the mesenchymal cell-origin tumours. The following antibodies were used: anti-alpha smooth muscle actin (α-SMA, mouse monoclonal, 1 : 500, M0851; Dako, Glostrup, Denmark) and anti-desmin (mouse monoclonal, 1 : 200, sc-23879; Santa Cruz Biotechnology, Dallas, TX, USA). Non-immune goat serum was used as a negative control. Deparaffinised slides were incubated in 3% hydrogen peroxide diluted with methanol and steamed with a 10 mmol/l citric acid buffer for antigen retrieval. After cooling at room temperature, the slides were incubated with a blocking solution (Life Technologies, Frederick, MD, USA) for 1 h at room temperature and the primary antibody overnight at 4 °C. The next day, the slides were incubated with a broad-spectrum secondary antibody and streptavidin-horseradish peroxidase conjugate (Life Technologies, Frederick, MD, USA). Diaminobenzidine (Vector Laboratories, Burlingame, CA, USA) was used for the visualisation of the antigen-antibody complex. The slides were counterstained with 10% haematoxylin. For the identification of fibrous stroma around the neoplastic cells, Masson’s trichrome staining was performed. The neoplastic spindle cells showed positivity for α-SMA (Figure 3A) and focal positivity for desmin (Figure 3B). The wavy collagen fibres around the stromal cells were stained blue by Masson’s trichrome staining (Figure 3C).

## DISCUSSION

The microscopic features of AMFB include neoplastic spindle or oval stromal cells with a moderate amount of eosinophilic cytoplasm aggregated around irregularly distributed blood vessels with bundles of collagen (Fletcher et al. 1992). The immunohistochemical profile of AMFB includes strong immunoreactivity to vimentin, as well as oestrogen and progesterone receptors (Fletcher et al. 1992; Qiu et al. 2014; Kass et al. 2019; Housseini et al. 2020; Shilpa et al. 2022). Immunoreactivity against smooth muscle actin and desmin has shown controversial results, with varying expression depending on the case (Qiu et al. 2014; Kass et al. 2019; Housseini et al. 2020; Shilpa et al. 2022).

In the present case, a mass over 10 cm in diameter completely replaced the uterine wall and caused the displacement of the abdominal organs, including the intestines, ovaries, and urinary bladder, requiring an immediate hysterectomy. Microscopically, the mass showed well-differentiated neoplastic perivascular spindle cells with alternating hypercellular and hypocellular areas with no mitotic figures. Abundant vascular structures surrounded with fibrous tissue were also detected. The neoplastic cells were immunoreactive to both α-SMA and vimentin, but negative to desmin. Strong and diffuse immunopositivity for vimentin in correlation with the histology confirmed the mesenchymal cell origin of the present tumour.

The differential diagnosis of AMFB includes myofibroblastoma, aggressive angiomyxoma, angiofibroma, haemangioma, and leiomyoma. Myofibroblastoma, which shares the same myofibroblastic cell origin as AMFB, is ruled out due to the absence of abundant vascular structures and a distinct perivascular growth pattern (Narasimhamurthy et al. 2023). AMFB should also be differentiated from aggressive angiomyxoma, which exhibits similar histological characteristics as AMFB (Fletcher et al. 1992; Sutton and Laudadio 2012), but is associated with a considerable risk of invasion and recurrence (Goh et al. 2022). Aggressive angiomyxoma is a rare, infiltrative stromal tumour, which has been reported in the vulvovaginal area, perineum, and pelvis (Sutton and Laudadio 2012; Angelico et al. 2022). The cut surface of an aggressive myxoma shows a tan-pink to tan-grey colour with a glistening and gelatinous appearance (Sutton and Laudadio 2012; Angelico et al. 2022), which were not shown in the present case. The absence of stromal mucin, alternating cellularity of the mass, and perivascular growth of AMFB are features in contrast to those of aggressive angiomyxoma, which exhibits hypocellularity with abundant myxoedematous stroma (Fletcher et al. 1992; Angelico et al. 2022; Goh et al. 2022; Shilpa et al. 2022). Occasional mucoid gland and nerve bundle entrapment, myxoid degeneration, and erythrocyte extravasation common in aggressive angiomyxoma were not detected in the present case (McCluggage and White 2000; Goh et al. 2022).

In addition to angiomyxoma, angiofibroma and haemangioma were ruled out due to the strong α-SMA immunoreactivity of the neoplastic cells. Only the focal positivity for desmin practically ruled out leiomyoma.

The histopathological origin of AMFB remains unclear, while some studies suggest that AMFB originated from the neoplastic proliferation of primitive mesenchymal cells in the subepithelial myxoid stromal zone in the lower female genital tract (Fletcher et al. 1992; Nielsen et al. 1996; McCluggage and White 2000). In the present case of uterine AMFB, the tumour may have developed from local chronic inflammation leading to granulation tissue hyperplasia, resulting in the abnormal proliferation of perivascular stem cells totally replacing the uterine wall.

Due to its low incidence, AMFB has not been thoroughly considered in the differential diagnoses by veterinary clinicians. Previous cases of AMFB had been classified as hemangiopericytomas or perivascular wall tumours in veterinary case reports, encompassing a wide range of tumours originating from cells of the perivascular wall and adventitia including myopericytoma, angioleiomyoma, AMFB, and angiofibroma (Hendrick 2017). Stout and Murray first suggested the term hemangiopericytoma describing tumours with “staghorn” vessels and perivascular growth of neoplastic cells, which turned out to be a non-specific pattern shared by numerous, unrelated lesions (Tsirevelou et al. 2010; Penel et al. 2012). Hemangiopericytoma as a diagnostic term is no longer preferred due to its uncertainty and broadness. A differential diagnosis of the tumours depending on histopathology and immunoreactivity is required for the precise diagnosis of perivascular wall tumours, in this case, AMFB.

Although AMFB had been reported as a small, well-demarcated tumour without a systemic effect, the patient, in the present case, exhibited signs of cardiac impairment such as elevated serum proBNP and lactate soon after the recognition of the enlarged tumour. The excessive enlargement of the uterine AMFB in the present case is suspected to be the cause of the heart failure by applying pressure on the ovarian arteries and caudal vena cava, therefore hindering the blood flow and loading burden on the heart. Impairment of the blood flow may have caused congestive heart failure and systemic hypoperfusion, leading to elevated serum lactate and proBNP. In addition to the mechanical risk factors on systemic circulation, AMFB is a tumour with abundant thin-walled blood vessels, and a considerable amount of blood flows within the tumour. A large AMFB in small animals (e.g., a domestic cat in the present case) would be a burden on the systemic blood circulation, and the surgical removal of a mass without proper preoperative preparation would result in a significant blood loss, leading to hypovolemic shock. Clinicians should be aware that the resection of such enlarged organs possessing an excess amount of blood without proper diagnosis would become life-threatening, as the hysterectomy performed in the present case caused cardiac arrest. If necessary, sufficient fluid resuscitation or providing an inotropic would be required to prevent hypovolemic shock from the excess blood loss.

To conclude, AMFBs should be considered for the differential diagnosis of uterine perivascular mesenchymal tumours for proper perioperative procedures.
